# GRB2 Promotes Malignant Behaviors of Breast Cancer by Modulating the Global Expression and Alternative Splicing Profiles in SK‐BR‐3 Cells Through Binding mRNA

**DOI:** 10.1002/cam4.70905

**Published:** 2025-05-19

**Authors:** Wei Liu, Yumian Huang, Lei Qiao, Le Chong, Luhua Xia, Aikeremu Abudurehaman, Hongyu Li

**Affiliations:** ^1^ Department of Breast Internal Medicine Xinjiang Medical University Affiliated Tumor Hospital Urumqi China; ^2^ Department of Breast and Thyroid Surgery The Affiliated Cancer Hospital of Xinjiang Medical University Urumqi Xinjiang China; ^3^ Department of Ultrasonography Xinjiang Medical University Affiliated Tumor Hospital Urumqi China; ^4^ Department of Nuclear Medicine Xinjiang Medical University Affiliated Tumor Hospital Urumqi China; ^5^ Postdoctoral Research Workstation of Tumor Hospital Affiliated to Xinjiang Medical University Urumqi China

**Keywords:** alternative splicing, breast cancer, GRB2, RBPs

## Abstract

**Purpose:**

The flexible protein GRB2 interacts with HER1–4 on the cell surface and regulates the development of tumor cells; meanwhile, it is also an RBP that plays an important role in post‐transcriptional regulation in eukaryotes, which affects every stage of mRNA synthesis, modification, splicing, and stabilization. Although some studies have found a connection between GRB2 and HER2‐overexpression breast cancer, highlighting the potential of GRB2 as a novel biomarker that stimulates tumor growth, limited data were available to elaborate on their interaction mechanisms.

**Methods:**

In this research, we found 396 different gene expressions between the Grb2‐knockdown group and the SK‐BR‐3 group by the RNA sequencing approach. After GRB2 was knocked down, 956 alternative splicing events occurred.

**Results:**

The fRIP‐seq results showed that GRB2‐binding reads were significantly enriched in the intron region, indicating that UUAGC and UUGGUUGG might be the binding motifs. An integration analysis of DEGs with the peak genes of fRIP‐seq revealed that 63 genes possess GRB2 binding sites on their mRNAs or antisense RNAs. By integration analysis of AS events with the peak genes of fRIP‐seq, 66 genes related to AS events were found.

**Conclusions:**

Above, these AS events may be regulated by GRB2 to promote the progression of HER2‐overexpression breast cancer.

## Graphical abstract

GRB2 as a flexible protein and an RBP that is involved in post‐transcriptional regulation in eukaryotes, influencing all stages of mRNA synthesis, modification, splicing, and stabilization.

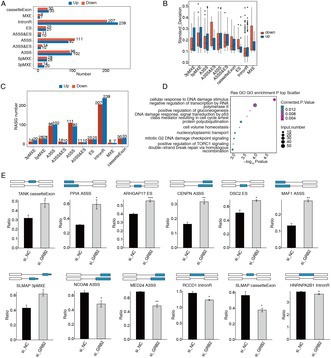

## 1 Introduction

In 2020, GLOBOCAN reported 2.3 million diagnosed cases and 685,000 fatalities [1, 2]; breast cancer presents a serious risk to women. The survival rate for patients has increased due to advancements in diagnosis and treatment strategies; however, the real number of new cases and fatalities has not decreased [3]; this has placed an enormous burden on the global economy [4]. Breast cancer is a highly heterogeneous malignant tumor [5, 6]; recurrence is inevitable and results in cancer‐related death [7, 8, 9].

A class of proteins called RNA‐binding proteins (RBPs) may attach to single‐ or double‐stranded RNA and interact with it to form ribonucleoprotein complexes [10, 11]. They are important participants in eukaryotic post‐transcriptional regulation, impacting all stages of mRNA synthesis, modification, splicing, and stabilization [11]. Growth factor receptor‐bound protein 2 (GRB2) is an RBP that possesses one SH2 domain and two SH3 domains [12, 13]. While the SH2 binds tyrosine phosphorylation sequences, the two SH3 directly form complexes with proline‐rich regions in other proteins [12, 14]. Four closely related transmembrane complex amino acid kinase receptors make up the ErbB receptor family: ErbB1–4 (HER1–4), all of them share 40%–50% of the same structure [15, 16]. HER2 is both a major cause and a highly valuable target in breast cancer. Silencing GRB2 inhibited cell proliferation and invasion [17]; LMTK3 kinase facilitated the invasion of breast cancer by inducing integrin β1 through GRB2‐mediated paths [18]; and MiR‐27b directly targeted CBLB and GRB2 to deactivate the MAPK pathways, which improved the response to paclitaxel [19].

In conclusion, we want to investigate the potential involvement of GRB2 in HER2‐overexpression breast cancer and offer a novel therapeutic approach.

## 2 Results

### 2.1 GRB2 Is Elevated and Has a Poor Prognosis in Breast Cancer Patients

**FIGURE 1:**
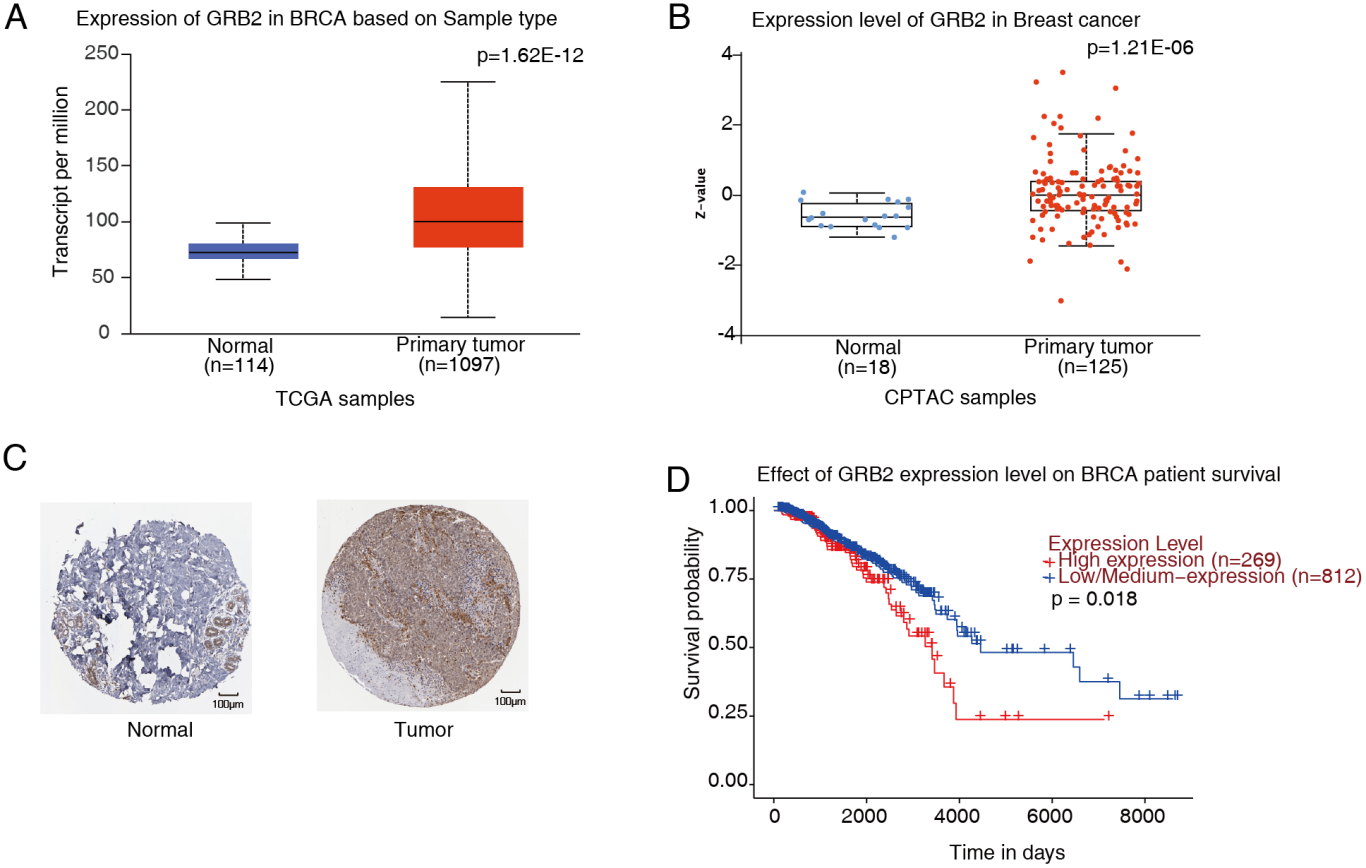
GRB2 is elevated and has a poor prognosis in breast cancer patients. (A) Box plot shows the mRNA of GRB2 in normal and tumor tissues from UALCAN. (B) Box plot shows the protein level of GRB2 in normal and tumor tissues. (C) GRB2 immunohistochemical demonstration from the HPA database. (D) The survival curve for GRB2 in breast cancer patients from UALCAN. BRCA, breast invasion carcinoma.

By utilizing the TCGA (The Cancer Genome Atlas Program) database accessible on UALCAN (https://ualcan.path.uab.edu/analysis‐prot.html), an increase in GRB2 mRNA levels was detected in breast cancer patients. Similar findings were observed when the GRB2 protein was analyzed in the CPTAC (https://pdc.cancer.gov/pdc/browse) database. Furthermore, an analysis of the HPA (The Human Protein Atlas) database indicated a notable increase in the expression of GRB2 in comparison to normal breast tissues (Figure 1A–C; Figure S1). The TCGA analysis demonstrated a correlation between a poor prognosis in patients and elevated levels of GRB2 (Figure 1D).

### 2.2 GRB2 Regulates Gene Expression in SK‐BR‐3 Cells

**FIGURE 2:**
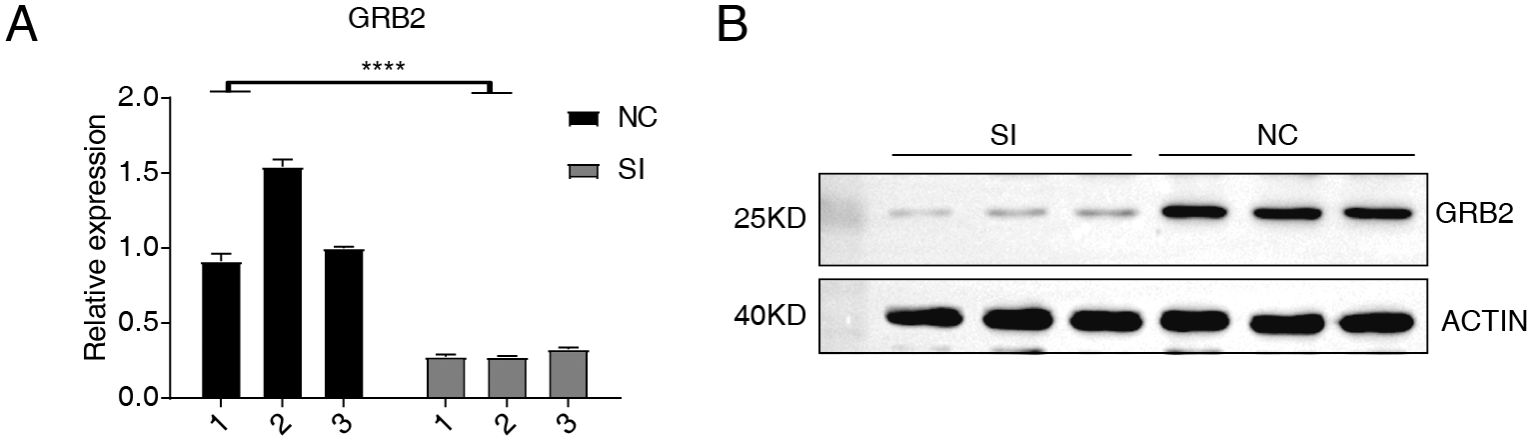
GRB2 knockdown in cells. (A) RT‐PCR shows expression of mRNA in cells. (B) Western blot demonstrates the effectiveness of the GRB2 knockdown. SI (1–3): GRB2 knockdown by small interfering RNA; NC (1–3): Control group.

**FIGURE 3:**
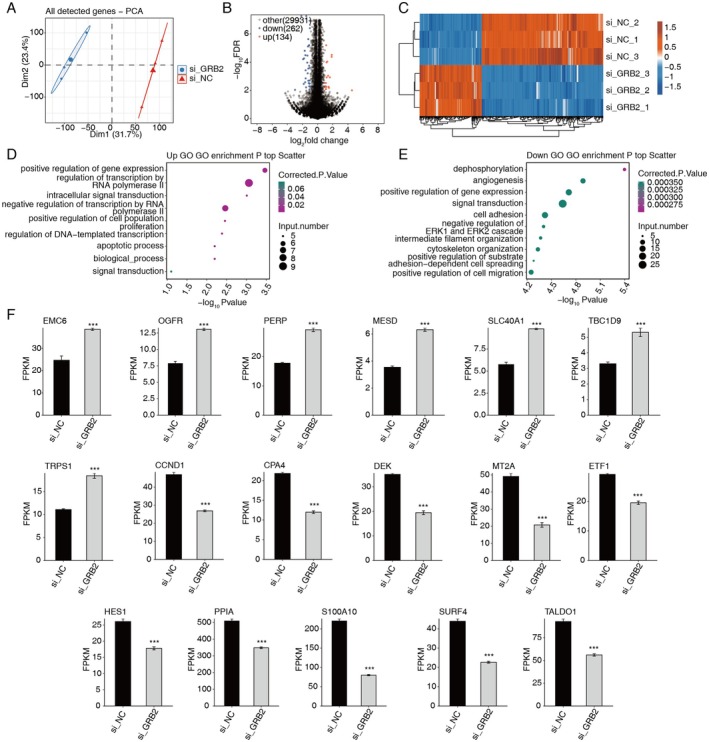
GRB2 regulates gene expression in cells. (A) PCA of samples after normalizing gene expression levels. (B) Volcano plot showing DEGs between si_GRB_2 and si_NC samples. (C) Heat map shows the level of DEGs. (D) Scatter plot displays the GO:BP of up‐regulated genes. (E) Scatter plot displays the GO:BP of down‐regulated genes. (F) Bar plot displays the pattern and difference of DEGs. ***p < 0.001.

SK‐BR‐3 cells, which overexpress HER2, were used to produce the GRB2 knockdown model (Figure 2A,B; Figure S2). Principal component analysis (PCA, Figure 3A) decreased the dimensionality of all the RNA‐seq data and identified 396 differentially expressed genes (DEGs) in the model, of which 134 were up‐regulated and 262 were down‐regulated (Figure 3B,C). The up‐/down‐regulated genes were enriched in the top 10 pathways (Figure 3D,E). The down‐regulated genes CCND1, CPA4, DEK, MT2A, ETF1, HES1, PPIA, S100A10, and TALDO1 may lead to the promotion of cancer (Figure 3F), while the up‐regulated genes EMC6, OGFR, PERP, MESD, SLC40A1, TBC1D9, and TRPS1 may be involved in suppressing cancer. GRB2 knockdown inhibited associated pathways, such as the NF‐κB and p53 pathway, depending on GSEA analysis of DEGs (Figure S3A,B). CCND1, PLAU, SERPINE1, CCL2, and S100A10, PROCR, RAP2B were noteworthy (Figure S3C,D).

### 2.3 GRB2 Regulates Gene AS in SK‐BR‐3 Cells

**FIGURE 4:**
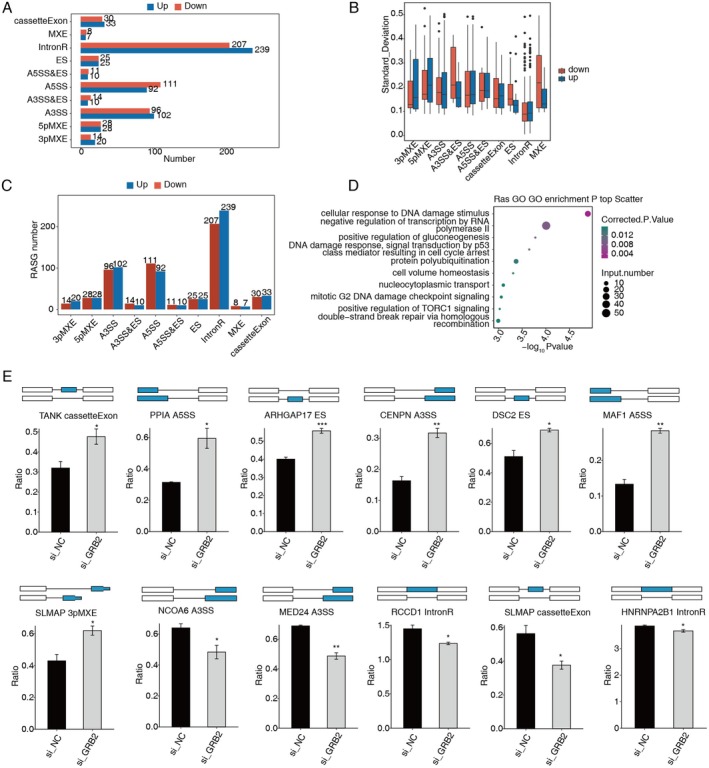
GRB2 regulates gene alternative splicing in SK‐BR‐3 cells. (A) Bar plot shows all RASEs in samples. (B) Boxplot shows the ratio of ASEs in samples. (C) Bar plot shows the gene number of ASEs. (D) Scatter plot exhibits the enriched GO:BP of the RASGs. (E) Bar plot shows the expression pattern and difference of genes. A3SS, alternative 3′ splice site; A5SS, alternative 5′ splice site; ASEs, alternative splicing events; MXE, mutually exclusive exons; RASEs, regulated alternative splicing events; RASGs, regulated alternative splicing genes. *p < 0.05; **p < 0.01; ***p < 0.001.

The RNA‐seq data's alternative splicing (AS) analysis revealed that a significant number of AS events occurred between the control and the GRB2 knockdown group (Figure 4A–C). While genes containing these events were identified, GO:BP revealed that these genes were enriched in the following procedures (Figure 4D). TANK, PPIA, ARHGAP17, CENPN, DSC2, MAF1, SLMAP, NCOA6, MED24, RCCD1, and HNRNPA2B1 that experience alterations must be specifically examined as they may be related to the advancement of breast cancer (Figure 4E).

### 2.4 GRB2 Binds to mRNAs in SK‐BR‐3 Cells

**FIGURE 5:**
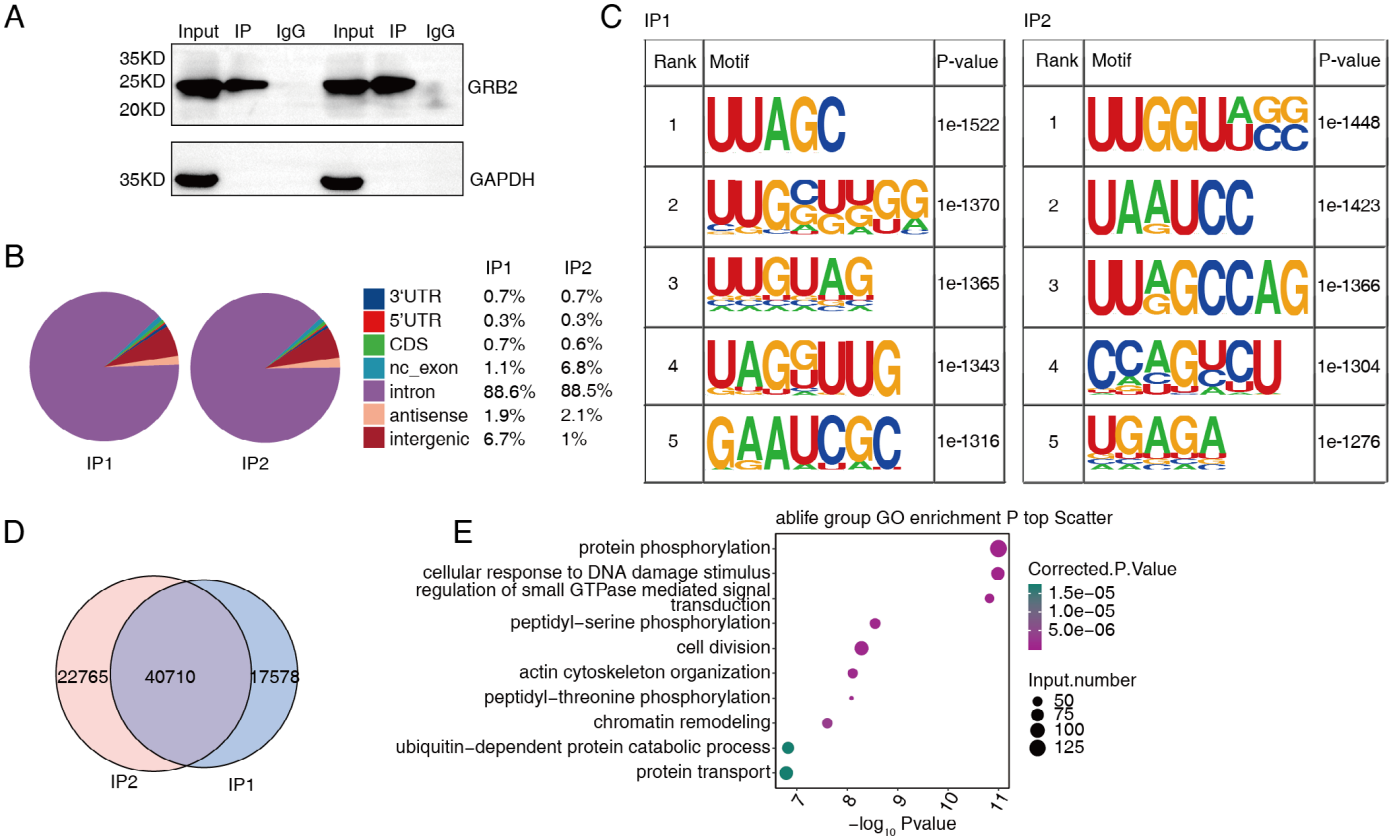
GRB2 binds to mRNAs in cells. (A) Western blot of GRB2 immunoprecipitates (IP) via an anti‐Flag monoclonal antibody. (B) Pie chart illustrates the genomic distribution of GRB2‐bound peaks. (C) Motif results reveal the presence of enriched motifs in the GRB2‐bound peaks. (D) Venn diagram illustrates the overlapped peaks. (E) Scatter plot exhibits the most enriched GO:BP results.

In the intron region, fraction RNA immunoprecipitation coupled with sequencing (fRIP‐seq) results showed a significant enrichment of GRB2‐bound reads (Figure 5B). UUAGC and UUGGUUGG may be the binding motifs shared by IP1 and IP2, with UUAGC ranking first and third in IP1 and IP2, and UAGGUUG ranking second and first in IP1 and IP2, among the highly typical motifs of binding peaks in the intron region of GRB2 (Figure 5C). The overlapping gene between IP1 and IP2 in two replicates is 40,710 (Figure 5D). Genes that bind to GRB2 and are associated with binding peaks are primarily abundant in pathways related to phosphorylation, cellular responses to DNA damage stimuli, chromatin remodeling, peptidyl‐serine phosphorylation, and cell division (Figure 5E).

### 2.5 Integration Exploration Between GRB2‐Bound RNAs and DEGs

**FIGURE 6:**
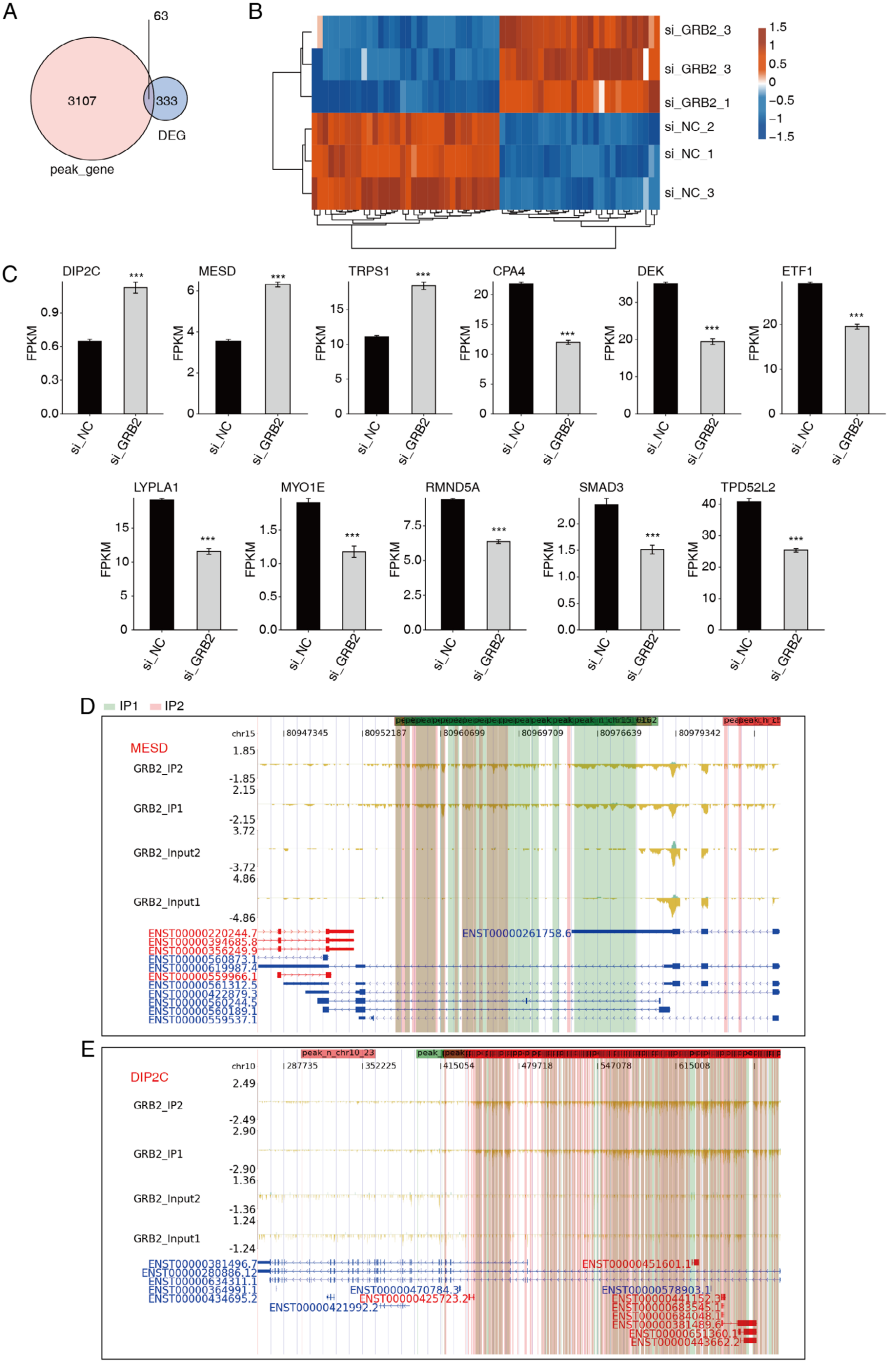
Integration exploration between GRB2‐bound RNAs and DEGs. (A) Venn diagram shows the overlapped genes between DEG and peaks genes. (B) Heat map shows the level of overlapped genes. (C) Bar plot shows the pattern and difference of DEGs.(D) GRB2‐binding peak genes of MESD. (E) GRB2‐binding peak genes of DIP2C. ***p < 0.001.

The GRB2 binding sites on the mRNAs or antisense RNAs of 63 genes with notable changes were found by integration analysis of the RNA‐ and fRIP‐seq data (Figure 6A,B). These genes consist of DIP2C, MESD, TRPS1, CPA4, DEK, ETF1, LYPLA1, MYO1E, RMND5A, SMAD3, and TPD52L2, whose expression may be related to breast cancer (Figure 6C–E).

### 2.6 Integration Exploration Between GRB2‐Bound RNAs and RASGs

**FIGURE 7:**
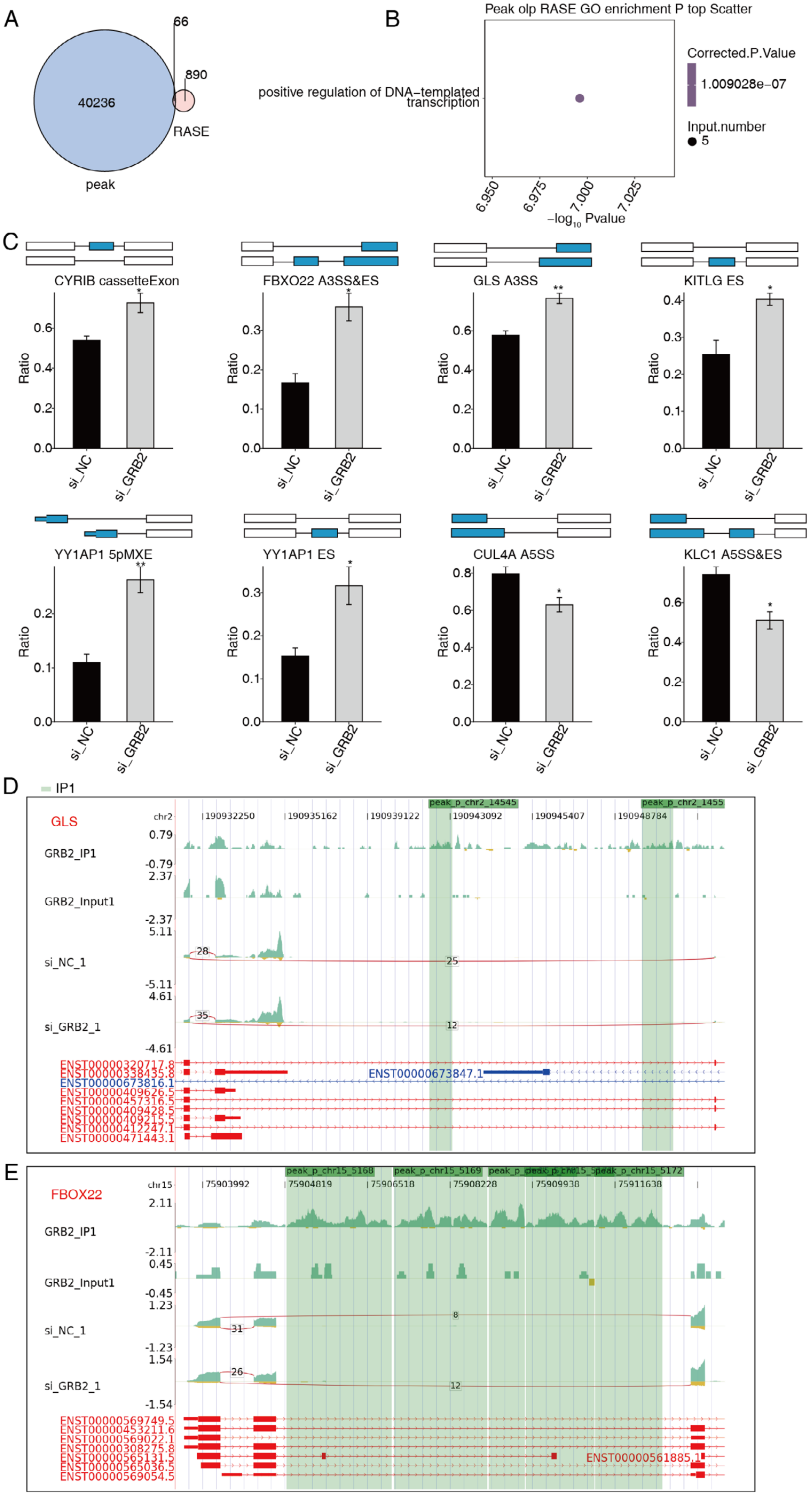
Integration exploration between GRB2‐bound RNAs and RASGs. (A) Venn diagram shows the overlapped peaks. (B) Scatter plot exhibits the enriched GO:BP results of peak genes. (C) Bar plot shows the expression pattern and difference of AS genes. (D) GRB2 regulates AS of GLS. (E) GRB2 modulates the process of AS for FBOX22. *p < 0.05; **p < 0.01.

The GRB2 binding sites on the mRNAs or antisense RNAs of 66 differential AS events were identified by integration analysis of GRB2‐bound RNAs and RASGs (Figure 7A). The majority of these genes were found to be enriched in positively regulated DNA‐templating transcriptional pathways (Figure 7B). In this pathway, GRB2 was shown to be capable of binding the mRNAs of CYRIB, FBXO22, GLS, KITLG, YY1AP1, CUL4A, and KLC1 and regulating their alternative splicing (Figure 7C–E).

## 3 Materials and Methods

### 3.1 siRNA Information

The siRNA duplexes were obtained from Gemma (RRID:SCR_008007, Suzhou, China). siNegative is a nontargeting control siRNA. 5′‐UUCUCCGA ACGUGUCACGU TT‐3′ (sense). The siRNA that targets GRB2 (siGRB2) is 5′‐GGUGGAUUAUCACAG AUCUTT‐3′ (sense).

### 3.2 Groups and Samples

SK‐BR‐3 cells (RRID:CVCL_0033, ZQ0080; Zhong Qiao Xin Zhou Biotechnology Co. Ltd., China) were divided into four groups: Control group (three samples) and GRB2 overexpression group (three samples), which were used to construct RNA‐seq libraries, and the data volume was 10 G/sample. GRB2 input group (two samples) and GRB2 IP group (two samples) were used for fRIP‐seq library construction; the data volume was 6 G/sample. The power calculation was *α* = 0.05.

### 3.3 Transfections and Cell Culture

SK‐BR‐3 cells (Zhong Qiao Xin Zhou Biotechnology Co. Ltd.) were a kind of female breast cancer cells expressing HER2 (3+), they were cultured at 37°C with 5% CO_2_ in DMEM with 10% FBS. The cells were transfected with 160 pmol/well siRNA for 5 min and 160 pmol/well Lipofectamine RNAiMAX Transfection Reagent for 5 min, mixed for 20 min. Finally, the mixture was added to the culture medium for examination by RT‐PCR and Western blot 48 h later. After confirming that there was no *Mycoplasma* infection by observing the cells grow well, the next test was performed.

### 3.4 Assessment of Gene Expression

The reverse transcription process was conducted at temperatures of 42°C for 5 min, 37°C for 15 min, and 85°C for 5 s using a thermocycler. The ABI Quant Studio 6 was used to perform qPCR. The concentration of each transcript was subsequently standardized to GAPDH and mRNA by the 2^−ΔΔCT^ method. Primers are provided in Table S1.

### 3.5 Western Blot

After lysing SK‐BR‐3 cells, a protease inhibitor cocktail was added to ice‐cold RIPA Buffer. The protein content was 1.33–1.98 mg/mL using the bicinchoninic acid (BCA) technique, with 10^6^ cells per sample (Table S2). The mixture was then set on ice for 30 min. These specimens were applied onto a 10% SDS‐PAGE gel, transferred onto 0.45 mm PVDF membranes, and subjected to boiling for 10 min in boiling water. Following an hour of blocking the PVDF membranes, they were then exposed to primary antibodies against GRB2 (RRID:AB_2263543, anti‐GRB2, 1:1000, antibody produced in rabbit, 10254‐2‐AP; Proteintech, China) and ACTIN (RRID:AB_764433, 1:5000, generated in rabbit; Proteintech) for an hour. Afterward, the samples were set in an incubator for 45 min with a secondary antibody that was linked to horseradish peroxidase (RRID:AB_219378, anti‐ rabbit, 1:10,000, SA00001‐2; Proteintech). The membranes were subsequently rendered visible by employing an improved ECL reagent and chemiluminescence.

### 3.6 RNA Extraction and Sequencing

Total RNA was extracted using TRIZOL. All of the samples exhibited RNA concentrations between 205.28 and 475.24 ng/μL; the *A*_260_/*A*_280_ between 1.857 and 1.972, which qualified for library building and satisfied standards for quality control (Table S3). The Illumina Library Preparation Kit was previously utilized for the purpose of generating customized RNA‐seq libraries; 1 μg of RNA for each sample was processed with RQ1 DNase to remove DNA. VAHTS mRNA capture beads were used to extract the mRNAs. Double‐stranded cDNA was created from fragmented RNAs. After end repair and tailing, they were ligated to VAHTS RNA Multiplex Oligos Set 1 for Illumina. Prior to sequencing, the ligated products underwent amplification, purification, quantification, and storage at −80°C. Strand‐specific sequencing is possible because the second cDNA strand, which is designated with dUTP, is not amplified.

The libraries for sequencing were generated and applied by the Illumina Novaseq Xplus instrument for 150 nt paired‐end sequencing.

### 3.7 RNA‐Seq Raw Data Clean and Alignment

Raw reads with a length greater than 2‐N bases were first removed. Subsequently, the FASTX‐Toolkit (Version 0.0.13) was employed to remove adaptors and low‐quality bases from the raw sequencing runs. The short readings with a length of fewer than 16 nucleotides were discarded. Afterwards, the clean reads were aligned to the GRCh38 genome using HISAT2 (RRID:SCR_015530, Kim et al. [20]), with a tolerance of four mismatches. The gene read number counting and FPKM computation were performed using uniquely mapped reads.

### 3.8 DEGs Analysis

The DESeq2 (RRID:SCR_000154) program from R Bioconductor (RRID:SCR_006442) was employed to determine DEGs. FC > 3/2 or ≤ 2/3 and *p* value less than 0.01 were the criteria to determine significant differential expression.

### 3.9 GSEA Analysis

GSEA is an analysis technique for microarray data with genome‐wide expression profiles. Gene pairs that have been predefined can be compared to identify functional enrichment. A collection of genes with similar roles, locations, pathways, or other characteristics is called a gene set. The ClusterProfiler package (RRID:SCR_016884, version 4.6.2) was used to perform GSEA. The FC in gene expression between the Mets and Primary groups was computed, and the |log_2_FC| change was used to produce the gene list. Next, we applied enriched GO:BP analyses based on GSEA.

### 3.10 Alternative Splicing Analysis

The ABLas pipeline was applied to evaluate the controlled RASEs and ASEs by the blind method. It detected 10 types of ASEs by splice junction reads. These included ES, A5SS, A3SS, MXE, IR, 5pMXE, 3pMXE, cassette exon, A3SS&ES, and A5SS&ES.

A paired *t*‐test was used to determine the *p*‐value of AS events. The *p*‐value cutoff (FDR of 5%) was classified as GRB2‐regulated ASEs.

### 3.11 Co‐Immunoprecipitation and Library Preparation

SK‐BR‐3 were washed twice with cold PBS and linked together for 10 min with 1% formaldehyde. A cross‐linking was stopped with 2.5 M glycine and rinsed twice with pre‐cooled PBS. Cold wash buffer, 400 U/mL protease inhibitor cocktail, and RNase inhibitor were used to lyse cells. After 30 min on ice, proceed to incubate RQ I in a heat block at 37°C until the concentration reaches 0.1 U/μL. The mixture was vigorously vibrated and centrifuged at 13,000 *g* for 15 min to remove cell debris.

Four micrograms of GRB2 antibody and a control IgG antibody (RRID:AB_2540050, AC005; ABclonal, China) were mixed with the supernatant overnight at 4°C. Protein A/G dynabeads were added to the immunoprecipitates and incubated for an additional 2 h at 4°C. The beads were rinsed six times with NT buffer after applying the magnet and discarding the supernatants. Resuspend beads in elution buffer. Agitate the mixture vigorously and heat it at 70°C for 30 min with a heat block to release immunoprecipitated RBP. Remove the magnetic beads from the separator and carefully transfer the liquid to a sterile 1.5 mL microfuge tube. Add 1.2 mg/mL Proteinase K to the 10% input and immunoprecipitated RBP‐containing RNA. At 55°C, incubate for 120 min. A phenol–chloroform–isopentyl alcohol reagent purified RNA.

The KAPA RNA Hyper Prep Kit was utilized for creating cDNA libraries following the guidelines, and we conducted 150 nt paired‐end sequencing by the Illumina NovaSeq Xplus.

### 3.12 Data Analysis

By matching reads to the genome using HISAT2, the comparison result of the duplicate was thrown out. Following that, we used Piranha and ABLIRC to do peak calls. The “ABLIRC” method was used to find the places where GRCh38 binds. How to perform a peak call: From the start of each chromosome, 5 bp was used as a window and a step. It stops being a peak when the total depth of the eight windows in a row is less than 4% and every gene has been randomly read 500 times. To do a significance analysis on the found peaks, the frequency of the deepest peak of each gene was added up. Any peaks with a maximum depth of at least 10 or a *p*‐value less than 0.05 were thrown out. After that, differences in abundance were analyzed based on where these peaks were found, using the input data as a standard. To find the best combined peak, the IP abundance had to be more than four times the input abundance. This can be changed as a parameter. The peaks helped find the IP target genes, and the HOMER software was used to see how the IP protein bound to the genes.

### 3.13 Functional Enrichment Analysis

The KOBAS 2.0 system has been used to identify GO and KEGG pathways in order to classify the function of DEGs. The hypergeometric test and the FDR adjusting technique were applied to ascertain the enrichment of the term.

### 3.14 Statistical Analysis

The mean standard deviation was utilized for presenting the data gathered from more than three independent experiments. The data were assessed using either a two‐way ANOVA for more than two groups or Student's paired *t*‐test for a two group test. *p*‐Value < 0.05 indicated statistical significance. All graphs were made using the GraphPad Prism software (Version 8.0; San Diego, CA).

## 4 Discussion

About 20%–25% of women diagnosed with breast cancer exhibited overexpression of HER2, a major target as well as a risk factor for recurrence. It is unclear why anti‐HER2 targeted therapy failed, despite the fact that targeted drugs have improved the survival of patients. We found that GRB2 was highly expressed in breast cancer tissues and was linked to a poor outcome for patients by using the TCGA, HPA, and UALCAN databases. But the way of interaction between Grb2 and HER2 is not well understood.

Few studies have been published, although GRB2 might take part in the post‐transcriptional regulation of target genes [21]. Integration analysis of RNA‐seq and fRIP‐seq data identified 63 genes that overlap, indicating that GRB2 may have an essential function in breast cancer [10]. The expression of EMC6, PERP, TRPS1, OGFR, SLC40A1, MESD, and TBC1D9 is increased following GRB2 knockdown. In gastric cancer cells, overexpression of EMC6 induces antitumor activity through the mitochondrial apoptosis pathway [22]. Oncogenic transformation of breast epithelial cells requires down‐regulation of PERP [23]. DCAF13 promotes cell proliferation by inhibiting PERP with ubiquitin [24]. TRPS1can stop the epithelial–mesenchymal transition [25]. SLC40A1 may result in resistance to cisplatin in ovarian cancer [26], promote prostate cancer growth [27] and reduce the hepatocellular carcinoma cells' ability to proliferate [28]. MESD, an inhibitor of many Wnt ligands, slowed the growth of prostate cancer in vivo [29] and decreased the invasion and migration of TNBC cells. Regardless of heterogeneity with respect to non‐TNBC patients [30], all of them had a downregulation of the TBC1D9 gene; and up‐regulation of TBC1D9 expression inhibited migration in colorectal cancer [31]. We speculate that GRB2 may aid breast cancer progress by decreasing the expression of these genes.

SURF4, DEK, ETF1, HSE1, CPA4, S100A10, TALDO1, MT2A, CCND1, PPIA, and S100A10 are the down‐regulated genes following GRB2 knockdown. Paclitaxel‐resistant breast cancer cells regained susceptibility after PPIA knockdown [32]. S100A10 acts as a metastasis‐promoting factor by encouraging invasiveness in breast cancer stem cells [33, 34]. RING1 induces apoptosis by suppressing the transcription of MT2A [35]. Greater CCND1 copy numbers are associated with greater histopathological grade and high proliferation, indicating that CCND1 may be involved in the formation of breast cancer [36]. Not only does the proto‐oncogene DEK induce an immunodeficient tumor environment with M2, but it has also been connected to poorer clinicopathological features and a worse prognosis for individuals with breast cancer [37]. We hypothesize that GRB2 may lead to the development of breast cancer by increasing the expression of these genes.

ARHGAP17, CENPN, MAF1, MED24, SLMAP, TANK, DSC2, PPIA, NCOA6, HNRNPA2B1, and RCCD1, which exhibited alterations in AS. The migration of cells is facilitated by VEGF‐long isoforms through the NRP1/ARHGAP17/Cdc42 network [38, 39]. CENPN may be an oncogene for breast cancer as it stimulates cell proliferation [40], as well as a potential target for immune checkpoint inhibitor therapy. CHI3L1suppresses CD8^+^ T cell activity and facilitates immunological escape from TNBC [41]. MED24 is related to the phenotype of breast cancer as well as a bad prognosis for patients [41]. Increased TANK‐binding kinase 1 levels, a protein implicated in EMT, strengthen the cells' resistance against triamcinolone acetonide's effects [42]. We hypothesize that GRB2 may affect AS, which may promote breast cancer.

Integration analysis of RNA‐seq and fRIP‐seq data demonstrated that 66 differential AS events were identified. GLS, YY1AP1, FBXO22, KLC1, CUL4A, KITLG, and CYRIB are changed by AS that may be linked to the emergence of breast cancer. In TNBC cell lines with dysregulation of the glutamine cleavage pathway, the loss of GLS leads to a severe inhibition of tumor growth [32] and it is related to tumor growth, metastasis, and the immune–tumor microenvironment [43]. YY1AP1 is an oncogenic driver [44], the high expression of YY1AP1 is linked to metastases and indicates a poor outcome overall [45]. FBXO22 has both pro‐tumor and anti‐metastatic properties [46, 47]. Anti‐tumor immune responses and treatment resistance in breast cancer cells have been linked to CUL4A [48]. CYRIB mediates pancreatic cancer's growth and signaling; downregulation of CYRIB promotes tumor cell migration, invasion, and EMT [49].

By identifying GRB2 binding mRNA and potential AS events in SK‐BR‐3 cells, we are able to get additional insight into the biological behavior of GRB2 and possible targets for treatment. In summary, GRB2 is connected with the advancement of HER2‐overexpression breast cancer. However, additional research is required to confirm GRB2's regulatory role in these genes.

## Author Contributions

**Wei Liu:** methodology (equal), validation (equal). **Yumian Huang:** methodology (equal), validation (equal). **Lei Qiao:** methodology (equal), validation (equal). **Le Chong:** investigation (equal), software (equal). **Luhua Xia:** investigation (equal), software (equal). **Aikeremu Abudurehaman:** formal analysis (equal), writing – original draft (equal). **Hongyu Li:** conceptualization (lead), data curation (lead), writing – review and editing (lead).

## Conflicts of Interest

The authors declare no conflicts of interest.

## Supporting information


Tables S1–S3.

Figures S1–S3.

## Data Availability

The data discussed in this publication are available under GEO Series accession number GSE276962 and GSE276963.
