# Supplementary material for: GRB2 Promotes Malignant Behaviors of Breast Cancer by Modulating the Global Expression and Alternative Splicing Profiles in SK‐BR‐3 Cells Through Binding mRNA
Source: Cancer Med. 2025 May 19;14(10):e70905. doi: 10.1002/cam4.70905 (PMC12086363; doi:10.1002/cam4.70905)
Supplement: Supplementary file 2 — Figures S1–S3. [file CAM4-14-e70905-s001.docx]

**
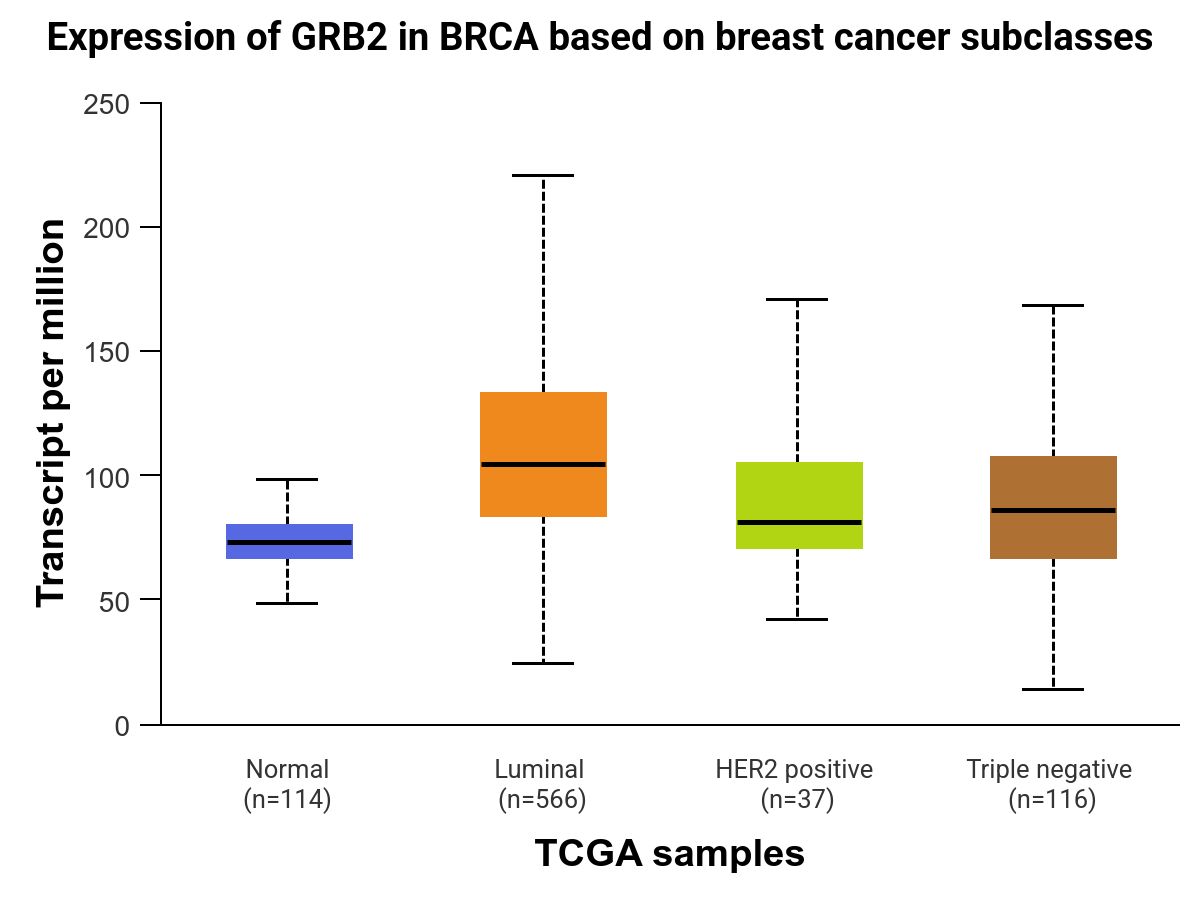
**

**FigS1. Expression of GRB2 in BRCA on breast cancer subclasses**

**
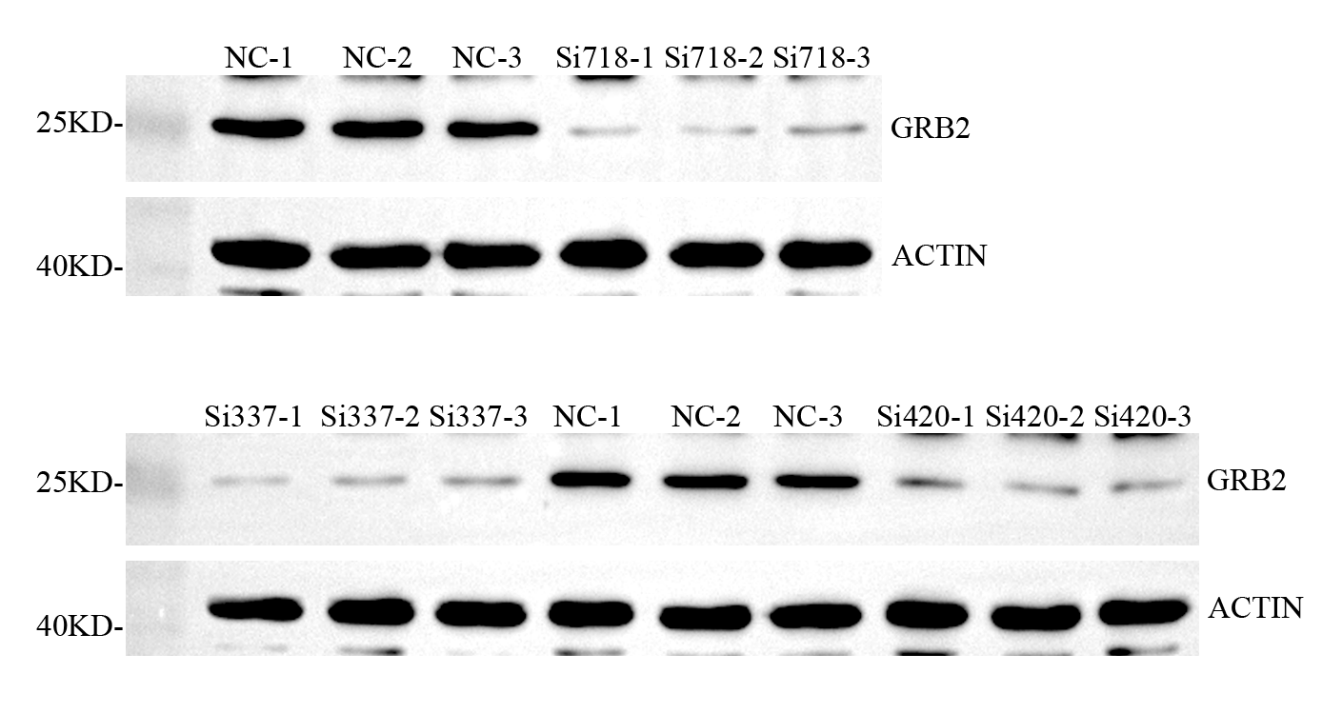
FigS2. Results of Western blot after GRB2 were knockdown by small interfering RNA.**

SI: small interfering RNA;NC:control group

**
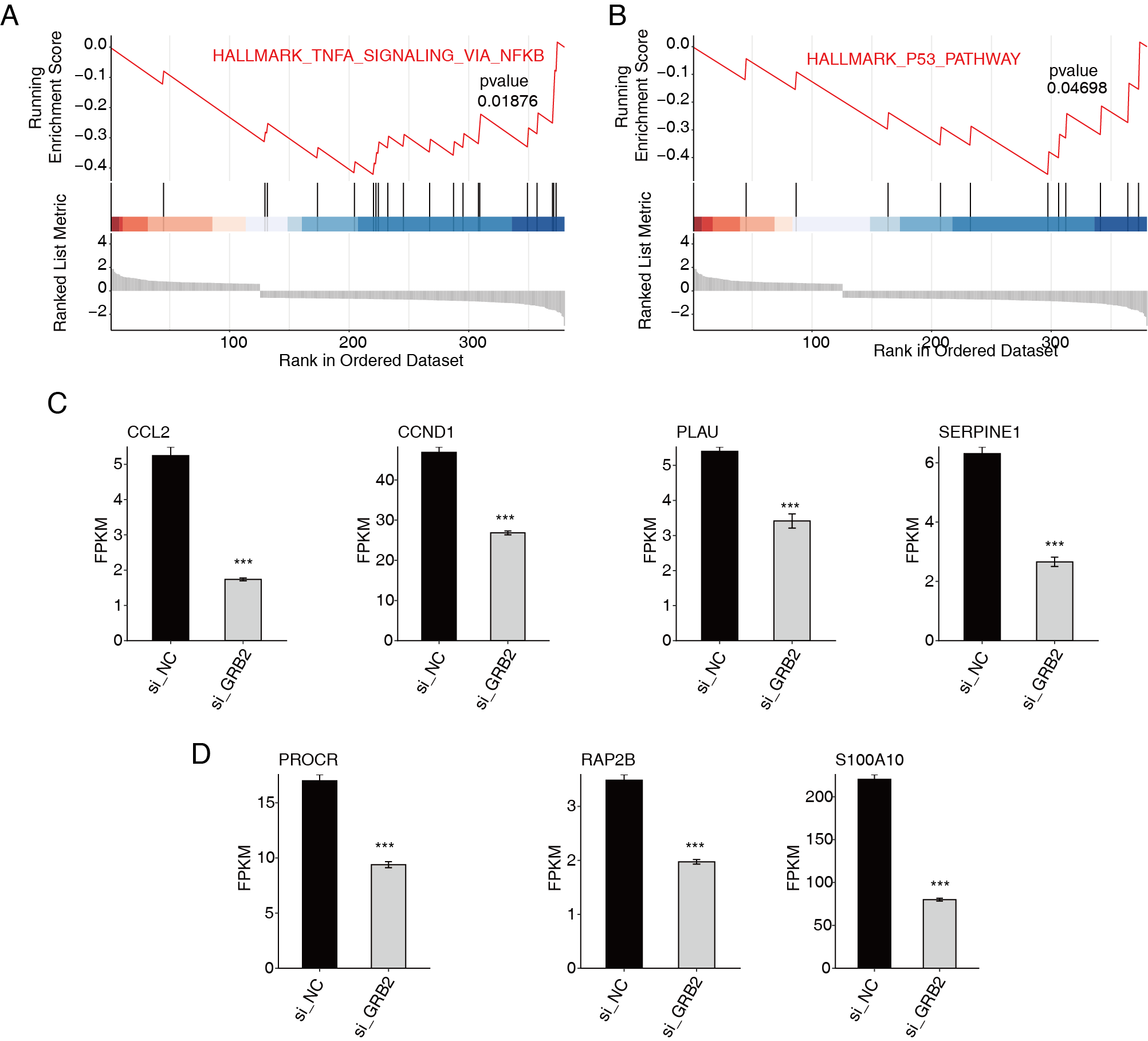
**

**FigS3. GSEA enrichment of DEGs the results of the analysis.**

1. TNFα signaling via NF-κB pathway integration for the visualization results.
2. P53 pathway integration for the visualization results.
3. Bar plot showing the expression pattern and statistical difference of DEGs from RNA sequencing. Error bars represent mean ± SEM. ***P-value < 0.001.
